# Association of pregnancy outcomes in women with type 2 diabetes treated with metformin versus insulin when becoming pregnant

**DOI:** 10.1186/s12884-020-03207-0

**Published:** 2020-09-04

**Authors:** Shu-Fu Lin, Shang-Hung Chang, Chang-Fu Kuo, Wan-Ting Lin, Meng-Jiun Chiou, Yu-Tung Huang

**Affiliations:** 1 Division of Endocrinology and Metabolism, Department of Internal Medicine, New Taipei Municipal TuCheng Hospital, New Taipei City, Taiwan; 2 Division of Endocrinology and Metabolism, Department of Internal Medicine, Chang Gung Memorial Hospital, Taoyuan, Taiwan; 3 College of Medicine, Chang Gung University, Taoyuan, Taiwan; 4 Division of Cardiology, Department of Internal Medicine, Chang Gung Memorial Hospital, Taoyuan, Taiwan; 5 Center for Big Data Analytics and Statistics, Chang Gung Memorial Hospital, Linkou, No.15, Wunhua 1st Rd., Gueishan Dist, Taoyuan City, 333 Taiwan; 6 Graduate Institute of Nursing, Chang Gung University of Science and Technology, Taoyuan, Taiwan; 7 Division of Rheumatology, Allergy and Immunology, Department of Internal Medicine, Chang Gung Memorial Hospital, Taoyuan, Taiwan; 8 Center for Artificial Intelligence in Medicine, Chang Gung Memorial Hospital, Taoyuan, Taiwan

**Keywords:** Pregnancy outcome, Metformin, Insulin

## Abstract

**Background:**

Metformin use in pregnancy is controversial because metformin crosses the placenta and the safety on the fetus has not been well-established. This retrospective study aimed to compare pregnancy outcomes in women with preexisting type 2 diabetes receiving metformin or standard insulin treatment.

**Methods:**

The cohort of this population-based study includes women of age 20–44 years with preexisting type 2 diabetes and singleton pregnancies in Taiwan between 2003 and 2014. Subjects were classified into three mutually exclusive groups according to glucose-lowering treatments received before and after becoming pregnant: insulin group, switching group (metformin to insulin), and metformin group. A generalized estimating equation model adjusted for patient age, duration of type 2 diabetes, hypertension, hyperlipidemia, retinopathy, and aspirin use was used to estimate the adjusted odds ratio (aOR) and 95% confidence interval (CI) of adverse pregnancy outcomes.

**Results:**

A total of 1166 pregnancies were identified in the insulin group (*n* = 222), the switching group (*n* = 318) and the metformin group (*n* = 626). The insulin group and the switching group had similar pregnancy outcomes for both the mother and fetus, including risk of primary cesarean section, pregnancy-related hypertension, preeclampsia, preterm birth (< 37 weeks), very preterm birth (< 32 weeks), low birth weight (< 2500 g), high birth weight (> 4000 g), large for gestational age, and congenital malformations. The metformin group had a lower risk of primary cesarean section (aOR = 0.57; 95% CI, 0.40–0.82) and congenital malformations (aOR, 0.51; 95% CI; 0.27–0.94) and similar risk for the other outcomes as compared with the insulin group.

**Conclusions:**

Metformin therapy was not associated with increased adverse pregnancy outcomes in women with type 2 diabetes as compared with standard insulin therapy.

## Background

Type 2 diabetes in pregnancy is a growing concern as the prevalence of type 2 diabetes in younger women is increasing and type 2 diabetes in pregnancy confers an increased risk of adverse pregnancy outcomes [1, 2]. Achievement of optimal glucose control before and during pregnancy is pivotal in order to minimize the occurrence of pregnancy complications [3]. Lifestyle modifications, including medical nutrition therapy and increased physical activity, are important in the management of type 2 diabetes; however, lifestyle interventions are usually insufficient to achieve glycemic targets in many patients and pharmacotherapy is frequently required [3, 4].

Insulin is the standard drug for the management of type 2 diabetes in pregnancy because it is effective for glycemic control and does not cross the placenta [3]. However, there are some barriers to insulin initiation, including injection phobia [5]. Metformin is an oral glucose-lowering agent that does not increase the risk of hypoglycemia and is the first-line pharmacological treatment for patients with type 2 diabetes [6–8]. The safety of metformin use in pregnancy has been reported in women with polycystic ovary syndrome (PCOS), gestational diabetes mellitus (GDM) and obesity [9–14]. However, the use of metformin during pregnancy remains controversial because metformin freely crosses the placenta and reaches concentrations in the fetus equivalent to those of the mother [15, 16]. USA guidelines suggest that metformin should be discontinued and insulin should be started as soon as possible when pregnancy occurs in patients with type 2 diabetes [3]. Current Australian guideline, however, is less restrictive [17].

In this study, we used national population cohort databases in Taiwan to compare pregnancy outcomes in women who received metformin or standard insulin treatment during pregnancy.

## Methods

### Data sources

The Taiwan National Health Insurance (NHI) is a single-payer system established in 1995 that provides universal medical care coverage to over 99% of the Taiwan residents (approximately 23.5 million people), including all citizens and foreigners living in Taiwan for more than 6 months [18]. This study analyzed data from the Taiwan NHI database, which was initiated in 1997 by the NHI Administration to facilitate research on health care. This database contains comprehensive information regarding patient demographic profiles (date of birth, sex, place of residence, and income level), detailed clinical information (diagnoses associated with inpatient and outpatient care and procedures), prescribed medications and surgeries, and fees incurred. Personal identification has been anonymized but kept consistent between NHI databases and other government-held datasets to facilitate accurate linkage [19].

The Taiwan Birth Registry dataset was also included in this study for the analysis of pregnancy outcomes. This data contains detailed birth characteristics of the mother and fetus, including method of delivery, gestational age, birth weight, Apgar score, congenital malformations, and stillbirth. In Taiwan, obstetricians have been required to report these data for the fetus at ≥20 weeks’ gestation to the Ministry of Health and Welfare since 1995. The Taiwan Birth Registry dataset has been shown to have high validity [20]. Both NHI and Birth Registry databases were approved to use for this study by the Health and Welfare Data Science Center, Ministry of Health and Welfare, Taiwan.

### Study cohort

**Fig. 1:**
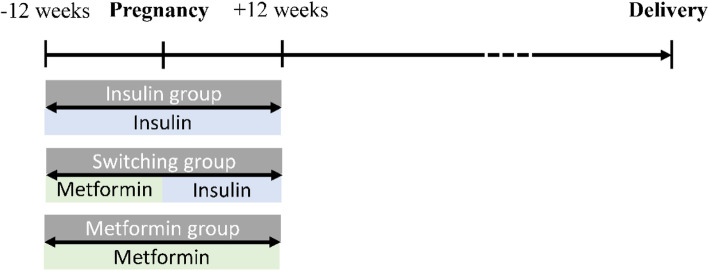
Three study groups according to glucose-lowering regimens

Data in the Taiwan NHI claim dataset and Birth Registry dataset between January 1, 2003 and December 31, 2014 were retrospectively analyzed. The case selection strategy to establish this study population was as following. Singleton pregnancies at ≥20 weeks of gestation in women aged between 20 and 44 years were identified in both databases. Pregnant women with preexisting type 2 diabetes (*International Classification of Diseases, Ninth Revision, Clinical Modification* [ICD-9-CM] codes: 250. × 0, 250.× 2) from outpatient and inpatient claims were identified. Women with a diagnosis of type 1 diabetes (ICD-9-CM codes: 250.× 1, 250.× 3) before pregnancy was excluded. Medications prescribed were identified using National Drug Codes in the NHI dataset. Three treatment groups were included in this study: 1) received insulin (insulin human, insulin lispro, insulin aspart, insulin glulisine, insulin detemir, or insulin glargine) and no oral antidiabetic drugs (metformin, sulfonylureas, dipeptidyl peptidase 4 inhibitor, thiazolidinediones, insulin secretagogues, or *α*-glucosidase inhibitors) before and after becoming pregnant (insulin group); 2) received metformin but no insulin before pregnancy and switched to insulin after becoming pregnant (switching group); and 3) received metformin but no insulin before and after becoming pregnant (metformin group) (Fig. 1). Oral hypoglycemic agents (e.g., sulfonylureas) were allowed to be administered concomitantly with metformin.

### Covariates

Covariates that could potentially affect pregnancy outcomes, including patient age, duration of type 2 diabetes, comorbidities (hypertension, hyperlipidemia, retinopathy) and aspirin use were identified and included in our analysis (see Supplemental Table S1). The proportion of subjects who used lipid-lowering agents (statins, ezetimibe or fibrates) and antihypertensive agents (angiotensin-converting-enzyme inhibitors, angiotensin receptor blockers, *β*-blockers, calcium channel blockers, thiazide diuretics, loop diuretics or potassium-sparing diuretics) was determined (see Supplemental Table S2). Our statistical analysis did not adjust for lipid-lowering agents and antihypertensive agents because dyslipidemia and hypertension were included as the covariates. Aspirin was included as one of the covariates because aspirin use in pregnancy has been shown to reduce the risk of preeclampsia [21]. Nephropathy and neuropathy were not included as covariates because of limited events.

### Study outcomes

Adverse pregnancy outcomes of the mother and fetus, including primary cesarean section, pregnancy-related hypertension, preeclampsia, preterm birth (< 37 weeks), very preterm birth (< 32 weeks), low birth weight (< 2500 g), high birth weight (> 4000 g), small for gestational age, large for gestational age, congenital malformations, Apgar score < 7 at 5 min and stillbirth were identified in the NHI and Birth Registry datasets.

### Statistical analyses

Patient characteristics were analyzed to determine the mean and standard deviation (SD), the median and interquartile range (IQR) for continuous variables, and the frequency for dichotomous variables. The crude incidence rates of adverse pregnancy events were calculated using the number of events divided by the total number of pregnancies for each group. Information for events involving fewer than 5 cases was not available because of the data-use policy for the NHI dataset.

Our primary analysis targeted women who had preexisting type 2 diabetes and were eligible for this study. We conducted two subgroup analyses, one limited to the primary cohort who had had type 2 diabetes for less than 3 years and the other limited to metformin users who received metformin as monotherapy. The risks of adverse pregnancy outcomes in the switching group and the metformin group were compared with the insulin group as the reference. The adjusted odds ratios (aOR) and 95% confidence intervals (CI) were computed using generalized estimation equations for each of the pregnancy outcomes adjusted for covariates using SAS (version 9.4; SAS Institute, Inc). The level of statistical significance in this study was set at *p* < 0.05.

## Results

### Maternal characteristics

There were 1166 pregnancies included for the primary analysis based on case selection strategy, 222 were in the insulin group, 318 were in the switching group and 626 were in the metformin group. The baseline patient characteristics of these three treatment groups are shown in Table 1.

**Table 1:** Study cohort characteristics

	Insulin Group (*n* = 222)	Switching Group (*n* = 318)	Metformin Group (*n* = 626)
*Age* at pregnancy, y, mean (SD)	34.48 (4.02)	34.05 (4.23)	33.51 (4.31)
*Age* at pregnancy, y, No. (%)			
20–29	29 (13.06)	48 (15.09)	129 (20.61)
30–34	86 (38.74)	124 (38.99)	258 (41.21)
35–39	90 (40.54)	123 (38.68)	189 (30.19)
40–44	17 (7.66)	23 (7.23)	50 (7.99)
*Duration of diabetes*, y, median (IQR)	3.65 (4.58)	3.10 (3.84)	1.49 (3.34)
*Baseline comorbidities, No. (%)*			
Hypertension	46 (20.72)	84 (26.42)	114 (18.21)
Hyperlipidemia	110 (49.55)	157 (49.37)	145 (23.16)
Retinopathy	24 (10.81)	21 (6.60)	26 (4.15)
Nephropathy	< 5 (< 2.25)^a^	< 5 (< 1.57)^a^	8 (1.28)
Neuropathy	5 (2.25)	< 5 (< 2.25)^a^	0 (0)
*Baseline medications, No. (%)*			
Aspirin	5 (2.25)	9 (2.83)	48 (7.67)
Statin	17 (7.66)	50 (15.72)	38 (6.07)
Ezetimibe	< 5 (< 2.25)^a^	< 5 (< 1.57)^a^	0 (0)
Fibrate	0 (0)	0 (0)	0 (0)
ACEI/ARB	7 (3.15)	18 (5.66)	26 (4.15)
Beta-blockers	< 5 (< 2.25)^a^	10 (3.14)	19 (3.04)
Calcium channel blockers	9 (4.05)	27 (8.49)	24 (3.83)
Diuretics^b^	< 5 (< 2.25)^a^	< 5 (< 1.57)^a^	12 (1.92)

*SD* standard deviation, *IQR* interquartile range, *ACEI* angiotensin-converting-enzyme inhibitor, *ARB* angiotensin II receptor blocker

^a^According to the data-use policy, information for events involving < 5 cases was not available

^b^Including thiazide diuretics, loop diuretics and potassium-sparing diuretics

### Pregnancy outcomes

The incidence of adverse pregnancy outcomes in each group is presented in Table 2. Using the insulin group as the reference, the risks of adverse pregnancy outcome in the switching group and the metformin group were computed using generalized estimation equations, adjusting for patient age, duration of type 2 diabetes, hypertension, hyperlipidemia, retinopathy, and aspirin use (Table 3). Compared with the insulin group, the switching group had a similar risk of primary cesarean section, pregnancy-related hypertension, preeclampsia, preterm birth, very preterm birth, low birth weight, high birth weight, large for gestational age, and congenital malformations. The metformin group had a lower risk of primary cesarean section (aOR, 0.57; 95% CI, 0.40–0.82; *p* = 0.002) and congenital malformations (aOR, 0.51; 95% CI, 0.27–0.94; *p* = 0.032) compared with the insulin group. The risks of other outcomes, including pregnancy-related hypertension, preeclampsia, preterm birth, very preterm birth, low birth weight, high birth weight, small for gestational age, large for gestational age, Apgar score < 7 at 5 min, and stillbirth were all similar in both the metformin group and the insulin group.

**Table 2:** Incidence of adverse pregnancy outcomes among women who received insulin, metformin to insulin switching or metformin therapy

Cohort	Insulin Group (*n* = 222)	Switching Group (*n* = 318)	Metformin Group (*n* = 626)
*Maternal outcome*, (%)			
Primary cesarean section	38.74	41.19	23.16
Pregnancy-related hypertension	24.77	29.87	20.77
Preeclampsia	16.67	20.13	13.26
*Fetal outcome*, (%)			
Preterm birth (< 37 week)	27.93	31.45	23.16
Very preterm birth (< 32 weeks)	4.50	3.77	3.99
Low birth weight (< 2500 g)	10.36	12.89	10.38
High birth weight (> 4000 g)	13.96	19.18	10.54
Small for gestational age	6.76	5.66	6.23
Large for gestational age	40.54	49.06	34.82
Congenital malformations	9.91	7.86	5.27
APGAR score < 7 at 5 min	< 2.25^a^	2.57	1.81
Stillbirth	3.60	2.20	2.72

^a^According to the data-use policy, information for events involving < 5 cases was not available

**Table 3:** Adjusted odds ratio of adverse pregnancy outcomes among women who received metformin to insulin switching therapy or metformin therapy as compared to insulin therapy

Cohort	Switching Group (*n* = 318)^a^	Metformin Group (*n* = 626)^a^
*Maternal outcome*		
Primary cesarean section	1.14 (0.80–1.63)	0.57 (0.40–0.82)
Pregnancy-related hypertension	1.26 (0.83–1.92)	0.92 (0.61–1.40)
Preeclampsia	1.24 (0.75–2.05)	0.92 (0.57–1.49)
*Fetal outcome*		
Preterm birth (< 37 week)	1.21 (0.84–1.75)	1.05 (0.69–1.59)
Very preterm delivery (< 32 weeks)	0.81 (0.33–2.02)	1.01 (0.42–2.46)
Low birth weight (< 2500 g)	1.32 (0.76–2.29)	1.30 (0.75–2.25)
High birth weight (> 4000 g)	1.47 (0.92–2.36)	0.91 (0.54–1.53)
Small for gestational age	–	0.98 (0.51–1.90)
Large for gestational age	1.39 (0.96–2.02)	0.99 (0.69–1.42)
Congenital malformations	0.75 (0.41–1.37)	0.51 (0.27–0.94)
Apgar score < 7 at 5 min	–	1.55 (0.31–7.67)
Stillbirth	–	0.82 (0.28–2.36)

^a^Adjusted for age, duration of type 2 diabetes, hypertension, hyperlipidemia, retinopathy, and aspirin use

### Additional analyses

Two secondary analyses were performed. First, we accessed the risk of study outcomes in women with type 2 diabetes of less than 3 years duration (Table 4). There were 154 patients (48.4%) in the switching group and 427 patients (62.6%) in the metformin group who had had known type 2 diabetes for < 3 years. Compared with the insulin group, the switching group had similar pregnancy-related risks for the mother and the fetus. The metformin group had lower risks of primary cesarean section (aOR, 0.50; 95% CI, 0.30–0.83; *p* = 0.008) and congenital malformations (aOR, 0.40; 95% CI, 0.17–0.93; *p* = 0.033) than did the insulin group. Comparable risk in the other pregnancy outcomes was observed in both the metformin group and the insulin group. These data are consistent with the primary findings.

**Table 4:** Adjusted odds ratio of adverse pregnancy outcomes in women with duration of type 2 diabetes < 3 years received metformin to insulin switching therapy or metformin therapy, as compared to insulin therapy

Cohort	Switching Group (*n* = 154)^a^	Metformin Group (*n* = 427)^a^
*Maternal outcome*		
Primary cesarean section	1.01 (0.59–1.74)	0.50 (0.30–0.83)
Pregnancy-related hypertension	1.65 (0.86–3.16)	1.08 (0.55–2.11)
Preeclampsia	1.39 (0.67–2.88)	0.97 (0.44–2.14)
*Fetal outcome*		
Preterm birth (< 37 week)	1.08 (0.60–1.95)	0.79 (0.43–1.47)
Very preterm delivery (< 32 weeks)	–	–
Low birth weight (< 2500 g)	1.26 (0.49–3.22)	1.27 (0.52–3.09)
High birth weight (> 4000 g)	–	–
Small for gestational age	–	–
Large for gestational age	1.43 (0.83–2.49)	0.79 (0.47–1.33)
Congenital malformations	0.60 (0.26–1.37)	0.40 (0.17–0.93)
Apgar score < 7 at 5 min	–	–
Stillbirth	–	0.23 (0.06–0.92)

^a^Adjusted for age, duration of type 2 diabetes, hypertension, hyperlipidemia, retinopathy, and aspirin use

To address the effects of metformin versus insulin treatment on pregnancy outcomes, we limited metformin users in the switching group and metformin group to those who received metformin as monotherapy. There were 273 patients (85.8%) in the switching group and 596 patients (95.2%) in the metformin group who received metformin as monotherapy (Table 5). In this analysis, the switching group had a similar risk of adverse pregnancy outcomes when compared with the insulin group. The metformin group had lower risks of primary cesarean section (aOR, 0.57; 95% CI, 0.40–0.82; *p* = 0.003) and congenital malformations (aOR, 0.47; 95% CI, 0.25–0.89; *p* = 0.021) compared with the insulin group. The risks of the other pregnancy outcomes were similar between the metformin group and the insulin group. The results of this analysis are also consistent with the primary findings.

**Table 5:** Adjusted odds ratio of adverse pregnancy outcomes among women who received metformin monotherapy in switching group and metformin group as compared to insulin therapy

Cohort	Switching Group (*n* = 273)^a^	Metformin Group (*n* = 596)^a^
*Maternal outcome*		
Primary cesarean section	1.06 (0.73–1.54)	0.57 (0.40–0.82)
Pregnancy-related hypertension	1.20 (0.78–1.86)	0.81 (0.53–1.23)
Preeclampsia	1.17 (0.70–1.95)	0.83 (0.51–1.34)
*Fetal outcome*		
Preterm birth (< 37 week)	1.07 (0.73–1.58)	0.97 (0.64–1.48)
Very preterm delivery (< 32 weeks)	0.51 (0.18–1.49)	0.93 (0.38–2.27)
Low birth weight (< 2500 g)	1.14 (0.64–2.04)	1.33 (0.76–2.34)
High birth weight (> 4000 g)	1.60 (0.98–2.60)	0.84 (0.49–1.45)
Small for gestational age	–	1.00 (0.51–1.97)
Large for gestational age	1.54 (1.05–2.25)	0.86 (0.60–1.23)
Congenital malformations	0.72 (0.38–1.35)	0.47 (0.25–0.89)
Apgar score < 7 at 5 min	–	1.48 (0.28–7.79)
Stillbirth	–	0.79 (0.26–2.39)

^a^Adjusted for age, duration of type 2 diabetes, hypertension, hyperlipidemia, retinopathy, and aspirin use

## Discussion

This investigation is the first nationwide population-based cohort study to assess the effects of different glucose-lowering regimens in women with preexisting type 2 diabetes on becoming pregnant. Our data demonstrate that metformin treatment is not associated with an increased risk of adverse pregnancy outcomes as compared with standard insulin treatment, whether continuing with metformin therapy or switching from metformin to insulin.

Our results show that many women with preexisting type 2 diabetes received metformin treatment during pregnancy. The popularity of metformin use during pregnancy in this study might result from the ease of use of this oral agent, the emergence of promising safety profiles for metformin use in pregnancy, and needle phobia [4]. The rising cost of insulin is one of the reasons supporting the use of metformin as an alternative to insulin during pregnancy [22–26]. However, NHI provides reimbursements for insulin and insulin is inexpensive in Taiwan [27]; therefore, affordability may not account for the frequent use of metformin in this study.

Our data reveal that metformin treatment did not increase the risk of adverse pregnancy outcomes. The safety of metformin use during pregnancy has been demonstrated in patients with GDM and with PCOS. In the largest study of women with GDM (*n* = 751) randomized to metformin or insulin treatment at 20–33 weeks of gestation, comparable pregnancy outcomes were observed between the two arms [12]; a follow-up study of their offspring reported that metformin-exposed children in the New Zealand cohort were larger at 7–9 years of age, although no such difference was seen in the Adelaide cohort [28]. In women with PCOS randomized to metformin or placebo treatment from the first trimester through to delivery, metformin therapy did not increase pregnancy complications over those seen with placebo [29]. However, metformin-exposed children were reported to have an increased prevalence of overweight or obesity at 4 years of age [30]. Interpretation of these follow-up data is difficult, given that postnatal factors might significantly influence obesity risk in children [22].

In the current study, the metformin group had a lower risk of congenital malformations compared with the insulin group. The mechanisms accounting for this observation are unclear. It has been reported that congenital malformations are proportional to increases in hemoglobin A1c during the first 10 weeks of pregnancy [31]. It is certainly tempting to think that women being managed with metformin might have less severe diabetes than those requiring insulin therapy, but the current databases do not provide such laboratory data in our population.

Our data reveal a higher risk of primary cesarean section in the insulin group than in the metformin group. Potential factors contributing to this observation are unclear and need to be clarified; however, maternal obesity might be involved. Maternal obesity is associated with higher rates of cesarean delivery [32]. Insulin therapy commonly results in weight gain, while metformin treatment has weight-neutral or weight-sparing effects [33, 34].

We found similar pregnancy outcomes between the switching group and the insulin group. The initiation and intensification of insulin therapy are usually difficult and challenging with respect to achieving tight glycemic control and preventing hypoglycemia [35]. Our results reveal that switching treatment from metformin to insulin did not increase the risk of adverse pregnancy outcomes over that of standard insulin treatment.

In this study, we found 58 pregnancies were treated with glyburide, another alternative oral glucose-lowering agent for pregnant women with type 2 diabetes. The limited number of glyburide users prevented further statistical analyses.

The present study has several strengths. First, the patient cohort was drawn from a nationwide database with a relatively large number of pregnant women with type 2 diabetes compared with prior reports [9–13]. Second, its design included active treatment comparison of clinically relevant drugs, including insulin and metformin. Both drugs are recommended for the treatment of pregnant women with type 2 diabetes. Third, the results are consistent across analyses of the primary cohort and two subgroups, supporting the validity of these findings. Fourth, our findings are in line with prior reports addressing the safety of metformin use in pregnancy [9–14].

This study has some limitations. First, pregnant women with type 2 diabetes treated with insulin may have poor glycemic control and a higher rate of comorbidities at the time of conception, which may lead to adverse pregnancy outcomes. We used surrogate indicators, including duration of type 2 diabetes and retinopathy, to adjust for the diabetes severity and also adjusted for comorbidities, including hypertension and hyperlipidemia. Second, this study did not assess pregnancy outcomes before 20 weeks of gestation because of the limit of the Taiwan Birth Registry dataset. However, a randomized placebo-controlled trial has reported that the use of metformin improves live-birth rates in women with PCOS [11]. Third, long-term outcomes for offspring were not evaluated in this study. Further studies to address this issue are mandatory. Fourth, the diagnoses of pregnancy-related hypertension and preeclampsia were based on ICD-9-CM codes, rather than based on clinical characteristics of individual patient in this study. Using ICD-9-CM codes may carry the risks of misclassification and underrepresentation. Of note, preeclampsia was diagnosed in 15.8% of pregnancies in this study, which is similar to prior reports showing10–14% women with type 2 diabetes had preeclampsia [36, 37]. Fifth, some covariates, such as glycemic control, pre-pregnancy body mass index and gestational weight gain were not included in our adjusted analyses because of the limited availability of NHI datasets. Sixth, propensity score matching was not performed to confirm our primary findings because of the relatively small sample size of each treatment group.

## Conclusions

In conclusion, our data reveal that metformin treatment for women with type 2 diabetes in pregnancy is not associated with an increased risk of adverse pregnancy outcomes compared with standard insulin therapy.

## Supplementary information

**Additional file 1: Supplemental Table S1.** Diseases and outcomes. **Supplemental Table S2.** List of medications.

## Data Availability

To secure the participants’ privacy, NHI research database and Taiwan Birth Registry data (the datasets of this study used) cannot be accessed outside the Health and Welfare Data Science Center, Ministry of Health and Welfare, Taiwan. The researchers must analyze these datasets at the Health and Welfare Data Science Center only.

## Abbreviations

PCOS: Polycystic Ovary Syndrome
GDM: Gestational Diabetes Mellitus
NHI: National Health Insurance
ICD-9-CM: International Classification of Diseases, Ninth Revision, Clinical Modification
SD: Standard Deviation
IQR: Interquartile Range
aOR: adjusted Odds Ratio
CI: Confidence Interval
