# Supplementary material for: Association of pregnancy outcomes in women with type 2 diabetes treated with metformin versus insulin when becoming pregnant
Source: BMC Pregnancy Childbirth. 2020 Sep 4;20:512. doi: 10.1186/s12884-020-03207-0 (PMC7487639; doi:10.1186/s12884-020-03207-0)
Supplement: Supplementary file 1 — Additional file 1: Supplemental Table S1. Diseases and outcomes. Supplemental Table S2. List of medications. [file 12884_2020_3207_MOESM1_ESM.docx]

Supplemental Table S1. Diseases and outcomes.

| **Diseases** | **ICD9-CM code** |
| --- | --- |
| Type 2 diabetes | 250.X0, 250.X2 |
| Type 1 diabetes | 250. X1, 250.X3 |
| Pregnancy-related hypertension | 642.3, 642.9, 642.4, 642.5, 642.6, 642.7 |
| Pre-eclampsia | 642.4, 642.5, 642.7 |
| Hypertension | 401, 402, 403, 404, 405 |
| Hyperlipidemia | 272.0, 272.1, 272.2, 272.3, 272.4 |
| Retinopathy | 362, 250.5, 379.23 |
| Nephropathy | 581, 585, 586 |
| Neuropathy | 337, 357 |
| **Entries of birth registry** |  |
| Primary cesarean section |  |
| Preterm birth (< 37 weeks) |  |
| Very preterm birth (< 32 weeks) |  |
| Low birth weight (< 2,500 g) |  |
| High birth weight (> 4,000 g) |  |
| Small for gestational age |  |
| Large for gestational age |  |
| Congenital malformations | 655 |
| Apgar score < 7 at 5 min |  |
| Stillbirth |  |

ICD-9-CM: International Classification of Diseases, 9^th^ revision, Clinical Modification

Supplemental Table S2. List of medications.

| **Drug Category** | **Non‐Proprietary Name** |
| --- | --- |
| Insulin | insulin human; insulin lispro; insulin aspart; insulin glulisine; insulin detemir; insulin glargine |
| Metformin | metformin; metformin & glibenclamide; metformin & glimepiride; metformin & repaglinide; metformin & pioglitazone; metformin & rosiglitazone; metformin & sitagliptin; metformin & saxagliptin; metformin & linagliptin; metformin & vildagliptin |
| Sulfonylureas | chlorpropamide; gliclazide; glimepiride; glimepiride & metformin; glipizide; glibenclamide; glibenclamide & metformin |
| DPP-4 inhibitor | linagliptin; linagliptin & metformin; saxagliptin; saxagliptin & metformin; sitagliptin; sitagliptin & metformin; vildagliptin; vildagliptin & metformin |
| Thiazolidinediones | pioglitazone; pioglitazone & metformin; pioglitazone & alogliptin; rosiglitazone; rosiglitazone & metformin |
| Insulin secretagogues | repaglinide; repaglinide & metformin; nateglinide |
| *α*-glucosidase inhibitors | acarbose; miglitol |
| Aspirin | acetylsalicylic acid |
| Statin | atorvastatin; rosuvastatin; simvastatin; pravastatin; fluvastatin; lovastatin; pitavastatin |
| Fibrate | fenofibrate; gemfibrozil; clofibrate; bezafibrate |
| Ezetimibe | ezetimibe; ezetimibe & simvastatin |
| ACEi | captopril; enalapril; quinapril; ramipril; lisinopril; imidapril; benazepril; benazepril & amlodipine; fosinopril; perindopril; perindopril & indapamide |
| Angiotensin receptor blockers | losartan; losartan & hydrochlorothiazide; eprosartan; valsartan; valsartan & hydrochlorothiazide; irbesartan; irbesartan & hydrochlorothiazide; c&esartan; c&esartan & hydrochlorothiazide; telmisartan; telmisartan & hydrochlorothiazide; olmesartan; olmesartan & hydrochlorothiazide; olmesartan & amlodipine; olmesartan, amlodipine & hydrochlorothiazide; azilsartan |
| *β*-blockers | carvedilol; metoprolol; bisoprolol; atenolol; labetalol; nebivolol; propranolol; oxprenolol; alprenolol; pindolol; timolol; nadolol; acebutolol; betaxolol |
| Calcium channel blockers | amlodipine; amlodipine & atorvastatin; felodipine; isradipine; nicardipine; nifedipine; nitrendipine; lacidipine; lercanidipine; benidipine; verapamil; diltiazem |
| Thiazide diuretic | hydrochlorothiazide; hydrochlorothiazide & losartan; hydrochlorothiazide & irbesartan; hydrochlorothiazide & c&esartan; hydrochlorothiazide & telmisartan & amlodipine; trichlormethiazide; cyclopenthiazide |
| Loop diuretics | furosemide; bumetanide; torsemide |
| Potassium-sparing diuretics | spironolactone; eplerenone; amiloride; amiloride & hydrochlorothiazide; triamterene & hydrochlorothiazide |

DPP-4: Dipeptidyl peptidase 4; ACEi: Angiotensin-converting-enzyme inhibitors
